# Temporal and spatial changes in the provision of mental health care during the COVID-19 pandemic in Germany: a claims-based cohort study on patients with severe mental disorders

**DOI:** 10.1007/s00127-023-02571-4

**Published:** 2023-10-13

**Authors:** Alexander Engels, Janine Stein, Steffi G. Riedel-Heller, Hans-Helmut König, Claudia Konnopka

**Affiliations:** 1 Department of Health Economics and Health Services Research, Center for Psychosocial Medicine, University Medical Center Hamburg-Eppendorf, Martinistr. 52, Building W37, 20246 Hamburg, Germany; 2 Institute for Social Medicine, Occupational Medicine and Public Health, University Medical Center Leipzig, Leipzig, Germany

**Keywords:** Germany, Mental health care, Mental health services, COVID-19 pandemic, Administrative claims, Secondary data analysis

## Abstract

**Purpose:**

Major lockdowns were imposed in Germany from March until May 2020 and from December 2020 until May 2021. We studied the influence of these lockdowns, the strain on intensive care units and the strictness of COVID-19-related containment strategies on the utilization of mental health care among patients with severe mental disorders.

**Methods:**

We used health insurance claims data to identify *n* = 736,972 patients with severe mental disorders shortly before the pandemic and *n* = 735,816 patients a year earlier. We applied entropy balancing to adjust for baseline differences by district. For a 12-month follow-up, we modeled monthly changes in utilization through meta-analytic models using both the COVID-19 stringency index and intensive care unit cases per 100,000 inhabitants as predictors. Our outcomes were changes in psychiatric hospital days and time treated by outpatient psychiatrists.

**Results:**

Psychiatric hospital days declined by at least 7.7% in all calender month during the pandemic. Peak reduction rates were observed in April (− 27.9%), May (− 22.3%) 2020 and January 2021 (− 18.3%). Utilization changes were associated with the stringency index and the second lockdown. Time treated by psychiatrists was shorter in April (− 16.2%) and May (− 11.5%) 2020 and in January 2021 (− 10.5%), which was partially offset by higher utilization in June and September 2020. These utilization changes were associated with the stringency index and the strain on intensive care units during both lockdowns.

**Conclusion:**

Hospitals did not maintain the level of utilization during the pandemic, while outpatient psychiatrists adapted more quickly, presumably due to digital and telemedical care.

**Supplementary Information:**

The online version contains supplementary material available at 10.1007/s00127-023-02571-4.

## Introduction

In Germany and other European countries, we observed a substantial increase in symptoms of depression and anxiety in the general population during the COVID-19 pandemic [1, 2], and even more in patients with preexisting conditions [3–6]. For the general population, the increase in mental health-related symptoms might be explained by occupational and financial changes [7], COVID-19-related fear [8] and increased loneliness due to contact restrictions [9]. In addition, the influence of the pandemic on mental health is mediated by how well people have adopted effective coping strategies such as positive thinking and social support [10, 11]. However, for patients with preexisting mental disorders, the burden was exacerbated by the decreased availability of formal psychiatric and psychosocial services due to restrictions. This aspect disproportionately affects patients who are most reliant on formal health services for support (i.e., patients with severe mental disorders). Unfortunately, few studies are available on how mental health care for patients with severe mental disorders was debilitated through the course of the pandemic [12].

Inpatient utilization of psychiatric services was reduced drastically during the first lockdown in Germany and again in December 2020 [13–17]. The first and the second lockdown in Germany comprised far-reaching restrictions (e.g., social distancing, contact restrictions, school closures, closure of retail and service companies), which were introduced from March 22 until May 5, 2020 and mostly reimposed from December 16, 2020, until May 2021. Even though the highest recorded death rate due to COVID-19 in Germany was recorded during the second lockdown, we know relatively little about the impacts of the second wave of infections on mental health care. Furthermore, all available studies concentrate on inpatient utilization, which means that we cannot assess whether outpatient mental health care may have compensated the limited availablility of inpatient services. For somatic disorders, there is some evidence that volumes of hospital admissions dropped more sharply than the number of physician consultations during the first lockdown [18]. One reason for the stronger decline of inpatient cases is that hospitals postponed elective operations to increase the capacity for COVID-19-related emergencies that would require mechanical ventilation. In addition, doctors’ offices can more easily reduce the risk of an infection for their patients by switching to remote telephone and video consultations, which were broadly and extensively used after the onset of the pandemic [19]. The question arises whether this applied to the mental healthcare sector as well, for example by implementing digital and telemedical services for outpatient mental care or by shifting patients from the inpatient to the outpatient sector. Therefore, we hypothesize that the utilization in the outpatient mental health care might have been less affected by the pandemic than inpatient mental health care for patients with severe mental disorders.

In this study, we want to analyze claims data to show trends in inpatient and outpatient utilization of mental health care during the first year of the pandemic comprising two lockdowns. We focus on patients with severe mental disorders, because this patient group is highly reliant on the formal health system and was strongly affected by the lack of its availability. Another aspect we want to analyze are regional differences in the course of the pandemic and how these affected changes in utilization. In an earlier analysis, we found substantial regional differences in the decline of case numbers in psychiatric and psychosomatic hospitals during the pandemic [13]. However, it remained unclear why hospital care in some regions was more drastically affected than in others, although an obvious explanation could be that those regions either implemented stricter measures or suffered from higher infection rates. In 2020, the western and southern regions of Germany as well as Saxony in the east were more strongly affected in terms of disease burden due to substantially higher infection rates [20]. Therefore, we want to explore whether proxies for the strictness of containment strategies and differences in intensive care unit cases can explain temporal and regional differences in changes in utilization.

Through the comparison of a control cohort that was observed before the pandemic and a pandemic cohort that was diagnosed shortly before the pandemic, we want to answer the following research questions:

1. How did the utilization of inpatient and outpatient mental health care for patients with severe mental disorders change during the first year of the pandemic in Germany?
2. Is there an association between the regional variation in utilization changes and the strain on intensive care units?
3. Is there an association between temporal variation in utilization changes and the strictness of containment strategies?

## Methods

### Study design and data sources

In this retrospective cohort study, we analyzed health insurance claims data of the “Wissenschaftliches Institut der AOK” (“WIdO”) for the period from January 1, 2018, to February 28, 2021. WIdO is the scientific institute of the AOK, which is the largest association of statutory health insurance companies in Germany. In total, the eleven autonomous companies of the AOK cover 26.8 million insurants (reference year 2019). This corresponds to about one third of the German population. The WIdO supplied us with data of all insurants who were continously insured at the AOK and had any diagnosis of a severe mental disorder by applying the following diagnoses of severe mental disorders treated in psychiatric hospitals or by psychiatrists: Schizophrenia (ICD-10: F20.x), schizoaffective disorder (F25.x), bipolar disorder (F31.x), severe depression (F32.2, F32.3, F33.2 or F33.3) or a personality disorder (F60.x). To determine the effects of the pandemic, we investigated a control cohort diagnosed between October 1, 2018, and February 28, 2019, and an exposed pandemic cohort diagnosed closely before the pandemic between October 1, 2019, and February 29, 2020. Subsequently, we tracked the utilitation of psychiatric inpatient and outpatient services for a 12-month follow-up period starting from March, 2019, (control cohort) to March, 2020 (pandemic cohort).

### Inclusion and exclusion criteria

We restricted the sample provided by the WIdO to patients with verified diagnoses of severe mental disorders. Given that physicians are legally obligated to encode ‘treatment diagnoses’ for accounting purpose in claims data, documented diagnoses may be less reliable than interview- or survey-based diagnoses [21]. Therefore, we focused on verified claims data diagnoses from university outpatient clinics, dayclinics, hospitals or mental health specialists (i.e., psychiatrists, psychotherapists and neurologists). Patients diagnosed by regular outpatient physicians were only included if the diagnosis was recorded as verified in two consecutive quarters. We excluded patients who died before the beginning of the follow-up period (*n* = 14,581) and patients with missing information in one of the relevant covariates (*n* = 925). For a detailed data flowchart, see supplemental Fig. 1.

**Fig. 1:**
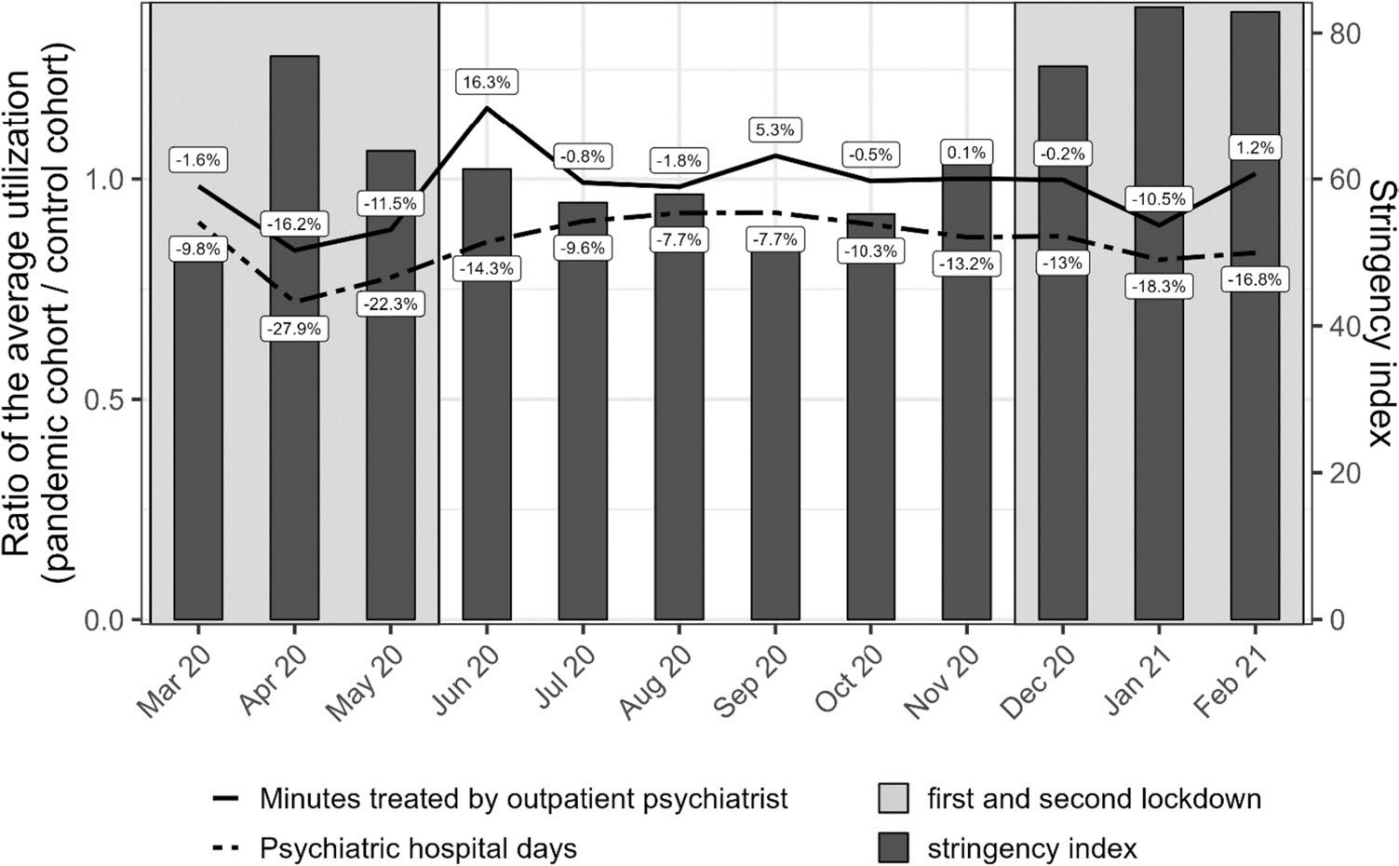
We compare the pandemic cohort’s utilization of mental health care during each calender month of the first 12 months of the pandemic with the control cohort’s utilization a year earlier. The two lines represent the relative change in utilization in both the time treated by outpatient psychiatrists and the number of psychiatric hospital days. The dark bars depict the strictness of the COVID-19-related containment strategies during that calender month as measured by the stringency index

### Aggregation at the regional level

We chose to aggregate the data by district and month, because we were mainly interested in predictors that varied regionally and across time (e.g., intensive care unit cases per 100,000 inhabitants or strictness of government policies). Hence, we used patient-level variables solely to account for potential baseline difference between the two cohorts. Germany consists of 294 rural districts and 107 urban districts. For each individual district, we employed entropy balancing [22, 23] to reweight the control cohort, so that the covariate moments of potential confounders (i.e., mean, variance and skewness) mirror the moments of the pandemic cohort in that particular district. Subsequently, we calculated the weighted average utilitation by cohort for each calender month and district.

### Outcomes and predictors of interest

We were interested in both inpatient and outpatient utilization of mental health care. Regarding inpatient care, we compared the number of hospital days due to a psychiatric discharge diagnosis (Fx.xx). Regarding outpatient care, we determined the time in minutes a patient was treated by psychiatrists. To that end, we took all psychiatric outpatient services into account that define a specific time requirement to be reimbursed. For psychiatric services, this approach should achieve reasonably high accuracy, because each completed 10 min of psychiatric consultations and interviews is reimburseable.

As predictors, we considered the burden on intensive care units and the strictness of containment strategies. The former was measured as the average number of intensive care unit patients per 100,000 inhabitants by calender month and district during the pandemic. The latter was quantified with the stringency index. The stringency index is a composite score that quantfies a country’s strategies to stop the spread of COVID-19 through closure of institutions (school or workplace closures) and containment (e.g., stay-at-home requirements and restrictions on mobility) [24]. Notably, the stringency index is only reported at a national level and therefore does not vary across regions.

### Covariates

As described, we employed entropy balancing for each district to adjust for confounding covariates. These covariates were determined based on the 9-month period before the index diagnosis. We considered sex, age on the date of diagnosis, the disease group (e.g., schizophrenia, severe depression or bipolar disorder) and the source of the diagnosis (i.e., mental health specialists, psychiatric hospitals, dayclinic, etc.). Regarding healthcare utilization, we controlled for the number of hospital days due to a psychiatric disorder (Fx.xx), the daily defined doses of antidepressants and antipsychotics, and the number of outpatient visits to psychiatrists. Somatic comorbidities were taken into account by calculating the 22 subscales of the medication-based comorbidity score [25]. However, we excluded rare comorbidities with few cases in our cohort. Otherwise, the inbalance between cohorts in entropy balancing may require assigning extremely high weights to individual patients, in order to achieve convergence, which might make the analysis more prone to outliers. Therefore, we excluded subscales if less than 30 patients in one of the cohorts were afflicted with the corresponding comorbidity in at least one district.

### Modeling approach

Given that all relevant predictors are related to COVID-19 and therefore only vary during the pandemic, we modeled changes in utilization at the district level as opposed to the absolute utilization in each cohort. First, we determined ratios of means (RoMs), which measure the relative difference in utilization between the pandemic cohort in a specific month and district and the control cohort a year earlier in the same district. RoMs greater than 1 would indicate that the utilization increased during the pandemic in a particular district and month, whereas RoMs below 1 would signify a reduction. Then, we employed meta-anayltic mixed-effect models to analyze the logarithmic RoMs. Considering that restrictions due to high infection rates persist to some extent across time, we assumed autoregressive temporal autocorrelation in the changes in utilization. In addition, we tested for spatial autocorrelation by calculating Moran’s I for each outcome by month [26]. The observed spatial autocorrelation was mostly close to zero (see supplemental Fig. 2). Therefore, we chose statistical models that assumed independence of the residuals of close districts.

**Fig. 2:**
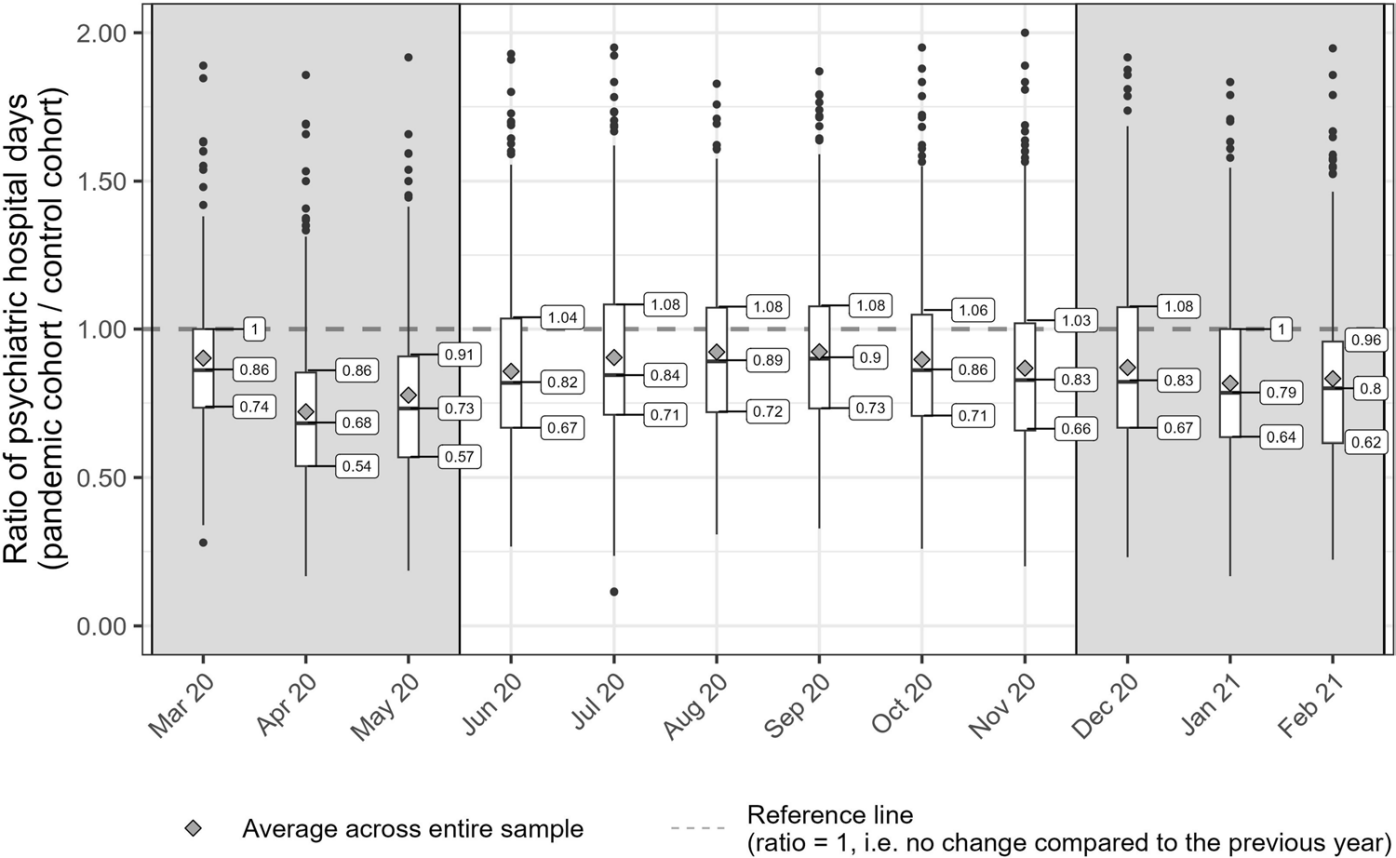
Distribution of the relative change in psychiatric hospital days across the 399 included districts We compare the pandemic cohort’s utilization of mental health care during each calender month of the first 12 months of the pandemic with the control cohort’s utilization a year earlier by district

## Results

We balanced for 12 of the 22 comorbities as measured by the comorbidity subscales (see supplemental Table 1 for the included and excluded subscales) and entropy balancing was successfully applied for 399 of the 401 German districts. Patients who lived in the German districts that did not converge (i.e., Bottrop and Salzgitter) were excluded (*n* = 4165). The final analysis included *n* = 735,816 patients with severe mental disoders in the control cohort and *n* = 736,972 in the pandemic cohort. At the district level, we included between a minimum of *n* = 604 from one of the smallest cities called Zweibrücken and a maximum of 50,542 patients from the capital city Berlin. This large range can be explained by both the substantial variation in the population size by district and the varying market share of the AOK as an insurer. The average sample size was *n* = 3691 patients per district. The control cohort had an unbalanced average age of 55.4 years (SD 17.1), 60.4% were female and most patients were included due to severe depression (46.1%), personality disorders (18.5%) or schizophrenia (17.1%). The pandemic cohort had an average age of 55.6 years (SD 17.0), 60.3% were female and most patients were again mostly included due to severe depression (46.6%), personality disorders (18.3%) or schizophrenia (16.9%). Supplemental table 2a-c shows a comparison of both cohorts with regard to a range of additional covariates including the region of residency, the source of the diagnosis, utilization during the preperiod and all comorbidity subscales.

To quantify the association of the pandemic and healthcare utilization, we determined RoMs by district and month. These RoMs already take the entropy balancing weights into account by dividing the average utilization of the pandemic cohort for each month by the weighted average utilization of the control cohort a year earlier. In Fig. 1, we weighted each RoM based on the total sample size of each district to estimate the monthly change in the utilization of psychiatric inpatient and outpatient care, respectively, at a national level.

The average number of psychiatric hospital days declined by at least 7.7% in all calender month during the pandemic when compared to the previous year. The highest reduction was observed during the lockdown periods in April (27.9%) and May (22.3%) 2020 and January 2021 (18.3%). For the average number of minutes treated by outpatient psychiatrists, we observed lower reduction rates across the entire follow-up period when compared to inpatient utilization. Substantial reductions in utilization were only observed in April (16.2%) and May (11.5%) 2020 and in January 2021 (10.5%). Contrary to the inpatient sector, we observed effects that offset this decline after the first lockdown in June and September of 2020, where the average utilization increased by 16.3% and 5.3%, respectively.

**Fig. 3:**
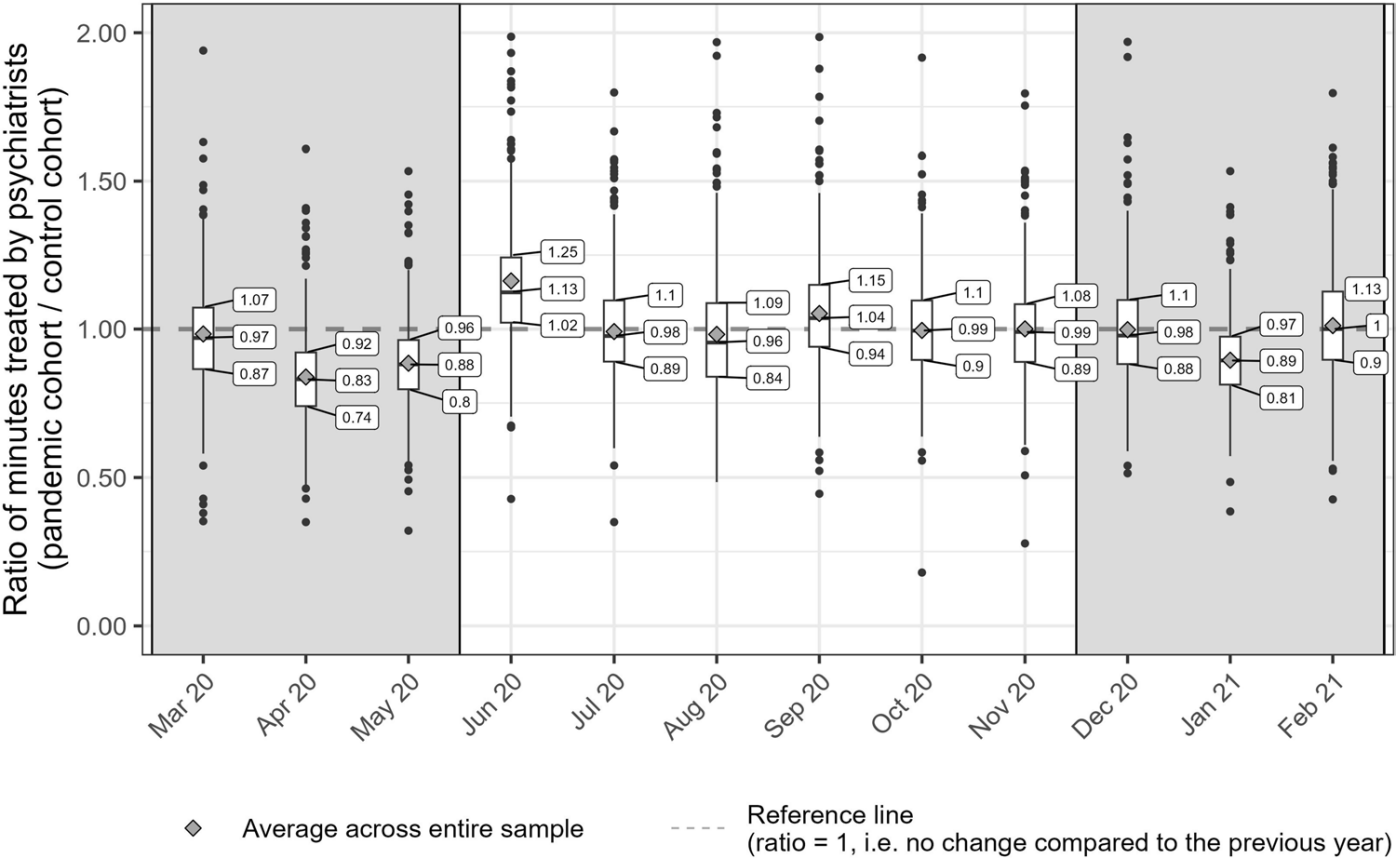
Distribution of the relative change in the time treated by outpatient psychiatrists across the 399 included districts. We compare the pandemic cohort’s utilization of mental health care during each calender month of the first 12 months of the pandemic with the control cohort’s utilization a year earlier by district

In Fig. 2, we used boxplots to show the distribution of RoMs for each calender month. We found substantial regional variation in the change in utilization during the pandemic. The largest interquartile range (IQR) in the RoMs for psychiatric hospital days was observed in December 2020 with 41%. This IQR means that the lower 25% of the districts (with an reduction of at least 33%) compared to the upper 25% of the districts (with an increase of at least 8%) differed by at least 41% in their absolute change in utilization. In contrast, we observed a low IQR in March 2020 with 26% and IQRs between 32 and 37% for all other calender months. The IQRs for the monthly changes in utilization for the number of minutes treated by psychiatrists were lower than for hospital days and ranged between 16% in January 2021 and 25% in August 2020 (Fig. 3).

In Table 1, we summarized the results of the meta-analytic models. We reported the results with and without covariates, but focused on the model with covariates in this paragraph. For psychiatric hospital days, we observed a significant negative effect for the stringency index, *β* = − 0.008,* t *= − 12.6,* p *< 0.001 and a positive effect for the second lockdown, *β* = 1.128,* t *= 2.97,* p *< 0.01. To clarify, the positive effect of the second lockdown does not indicate that the utilization of psychiatric hospitals increased during the second lockdown, but that the utilization was higher than expected in view of the high stringency of the containment strategies during this period. We found no significant interaction between intensive care unit cases per 100,000 inhabitants and the first, *β* = − 0.015,* t *= − 1.39,* p *= 0.17, or the second lockdown, *β* = − 0.008,* t *= − 1.35,* p *= 0.18. Most covariates had no signifcant effect (see supplemental table 3a). However, we found an association with the average age of the district’s population, *β* = − 0.026,* t *= − 3.16,* p *< 0.01.

**Table 1:** Results of the meta-analytic model to explain changes in the average utilization

		Without covariates		With covariates	
Outcome	Predictor	*β*	std. error	*β*	std. error
Inpatient sector: ratio of psychiatric hospital days	Intercept	0.298***	(0.037)	0.028	(0.315)
	Lockdown 1 (L1)	− 0.058***	(0.013)	− 0.506	(0.358)
	Lockdown 2 (L2)	0.182***	(0.030)	1.128**	(0.379)
	Stringency index	− 0.008***	(0.001)	− 0.008***	(0.001)
	Intensive care unit cases per 100,000 inhabitants	− 0.001	(0.004)	0.000	(0.004)
	Intensive care unit cases per 100,000 inhabitants in L1	− 0.015	(0.011)	− 0.015	(0.011)
	Intensive care unit cases per 100,000 inhabitants in L2	− 0.012*	(0.006)	− 0.008	(0.006)
Outpatient sector: ratio of minutes treated by psychiatrists	Intercept	0.300***	(0.019)	0.591***	(0.141)
	Lockdown 1 (L1)	− 0.120***	(0.008)	− 0.216	(0.214)
	Lockdown 2 (L2)	0.135***	(0.016)	− 0.008	(0.220)
	Stringency index	− 0.005***	(0.000)	− 0.005***	(0.000)
	Intensive care unit cases per 100,000 inhabitants	0.002	(0.002)	0.002	(0.002)
	Intensive care unit cases per 100,000 inhabitants in L1	− 0.015**	(0.006)	− 0.015*	(0.006)
	Intensive care unit cases per 100,000 inhabitants in L2	− 0.017***	(0.003)	− 0.018***	(0.003)

*N* = 1,472,788, standard error (std. error), the model estimates the coefficients using logarithmic ratios of means as an outcome, in the model with covariates we included the following control variables: German Index of Social Deprivation, average age of the district, population density, hospital density, general practitioner and psychiatrist density. We included an interaction effect with each lockdown for each control variable, respectively, to allow for deviating effects in the two lockdown periods

For the minutes treated by psychiatrists, the model showed a significant negative association with the stringency index, *β* = − 0.005,* t *= − 14.56,* p *< 0.001. Furthermore, it revealed a significant interaction between intensive care unit cases per 100,000 inhabitants and the first, *β* = − 0.015,* t *= − 2.488,* p *< 0.05 and the second lockdown, *β* = − 0.018,* t *= − 5.422,* p *< 0.001. Most covariates had no signifcant effect (see supplemental table 3b). However, we found an association with the district’s general practitioner density, *β* = − 0.002,* t *= − 2.866,* p *< 0.01.

Considering that these beta coefficients quantify the association between the predictor and the logarithmic ratio of means, it can be difficult to judge how exactly the expected changes in utilization are affected by the predictors. Therefore, we provide supplemental Figs. 3 and 4 to illustrate the models’ predictions for each calender month—based on the lockdowns, the stringency index and a realistic range of values for intensiv care unit cases per 100,000 inhabitants.

## Discussion

In this study, we give an overview of the state of mental health care for patients with severe mental disorders during the first year of the COVID-19 pandemic. Contrary to existing studies that were restricted to inpatient cases [13, 15] or emergency hospital admissions [14, 17], we compared a large cohort of patients diagnosed across sectors before the pandemic with a cohort that was diagnosed a year earlier. Subsequently, we controlled for various patient characteristics to obtain a reliable assessment of how utilization changed between the two comparable cohorts during the pandemic. This is the first study to assess the pandemic’s associtation with the utilization of psychiatrists in outpatient care and to model changes in utilization at a district level.

In the inpatient sector, we found a noticable decline of hospital days due to psychiatric disorders across the entire 12-month follow-up. Peak reduction rates were observed at the beginning of the pandemic in April (− 27.9%) and May 2020 (− 22.3%), because hospitals yet had to adapt to an unprecended and unclear situation. During the first lockdown, hospitals postponed or suspended elective surgeries and procedures to expand intensive care units and ventilator capacities for COVID-19 patients. Non-acute medical departments were commonly repurposed for the management of COVID-19 patients. In addition, dayclinics were often closed until appropriate hygiene and safety measures (e.g., special hygiene measures, restrictions in personal therapeutic contact and distance regulations) were implemented. Nevertheless, we assume that the reductions in January 2021 (− 18.3%) and February 2021 (− 16.8%) suggest that the overall impact of the second lockdown has been stronger than the first lockdown due to its longer duration from December 2020 to May 2021.

In the outpatient sector, patients often canceled doctor appointments, check-ups and preventive consultations to avoid the risk of an infection [27, 28], but overall we observed less pronounced consequences for the utilization of services. The time treated by psychiatrists declined in April (− 16.2%) and May 2020 (− 11.5%), but over the remaining study period, we found almost pre-pandemic utilization rates—except for a noticable drop during the second lockdown in January 2021 (− 10.5%). One reason for the relatively mild consequences for outpatient care could be that digital and telemedical care services were quickly implemented and increasingly used throughout the pandemic [29, 30]. This would highlight the relevance and the potential advantages of telemedicine as a potential coping strategy.

Apart from the large temporal variation, we also observed substantial regional variation in the changes in utilizations in both sectors. The utilization of the 25% of the districts with the highest utilization of hospital care throughout the pandemic declined at most by − 14% over the 12 months of follow-up period, whereas the 25% with the lowest utilization declined by up to − 46%. This fluctuation across districts was less pronounced in the outpatient sector, where the 25% of the districts with the highest utilization of psychiatrists declined at most by − 8%, while the 25% with the lowest utilization declined by up to − 26%.

We identified some factors that could explain the temporal and the regional variation. First, we found a relatively strong association between the decline in utilization and the stringency index [24] in both sectors. The stringency index quantifies the governmental restrictions on mobility and social contacts during the pandemic. Therefore, it may not be surprising that the utilization of medical services was affected when public life was restricted or brought to a halt. In addition, we found a positive effect of the second lockdown on inpatient utilization. This positive effect of the second lockdown does not indicate that the utilization of psychiatric hospitals increased during the second lockdown, but that the utilization was higher than expected in view of the high stringency of the containment strategies during this period. So both lockdowns led overall to a decline in utilization, but the reduction was less pronounced during the second lockdown. Probably, psychiatric hospitals could better prepare for the second than for the first lockdown and develop coping strategies such as hygiene concept to reduce the probability of infections or use telemedicine, where applicable. The third factor was the strain on intensive care units, which was measured as intensive care unit cases per 100,000 inhabitants and variied at a district level. Regarding outpatient care, we found a stronger decline in utilization in districts with a high strain on intensive care units during the lockdown periods, but we did not observe any effect on psychiatric hospitals days. Contrary to the imposed restrictions that are directly enforced by institutions, we assume that the strain on intensive care units might primarily have reduced a patient’s willingness to utilize services through increased fear of a COVID-19 infection. It seems plausible that this fear might be less influential in the inpatient sector, because patients in psychiatric hospitals tend to experience an acute and severe crisis that can not be postponed despite the increased risk of an infection. This explanation would also be supported by an earlier analysis, where we found smaller reductions in case numbers for patients with more severe disorders (e.g., schizophrenia) when compared to more common mental disorders (e.g., affective disorders) [13].

On the other hand, based on health insurance claims data we cannot draw a clear conclusion of the reasons for decreased utilization. We cannot answer the question if patients could not be treated in hospitals (as bed were blocked) and/or if they did not want to be treated (due to their fear of infections). Utilization of outpatient care did not decline as much (nor as long) as inpatient care. Probably, the outpatient care sector showed to be more resilient during the lockdown, as measures such as telemedicine could be applied. In this case, the outpatient care sector should be more accessible for suitable patients, e.g., by supporting integrated care models or inpatient-equivalent treatment where patients are treated by a multidisciplinary team at home, reducing waiting times, simplifying the reimbursement of outpatient treatments or further applying digital health applications.

Furthermore, the question arises whether some patients could successfully be transferred from inpatient care to outpatient care during the pandemic. In case of patients with potential overuse of inpatient care and excessively long hospital stays, in parts favored by the current German remuneration system, this might even be beneficial. On the other hand, outpatient care might need a planned and managed expansion to sufficiently substitute or supplement inpatient care and provide potential advantages for the patients' health in the long run. Future research might focus on the question whether the pandemic has given a positive impulse to promote outpatient care where possible.

### Strengths and limitations

We compared two large cohorts with more than 735,000 patients with severe mental disorders. Considering that we used health insurance claims data from the scientific institute of the AOK, we drew from a pool of about 27 million insurants. Therefore, we utilized claims data of about a third of the German population. In addition, we reweighted the control cohort in each district via entropy balancing. This means that we obtained relatively unbiased estimates of the changes in utilization due to the pandemic, because control and pandemic cohort were comparable in a large variety of control variables in each district. Another strength is that we modeled both the changes in the inpatient and the outpatient sector and developed a methodological rigor approach to explain these changes with COVID-19-related predictors.

Nonetheless, some limitations are worth noting. First, health insurance claims data lack information on, e.g., the severity of the disease based on psychopathological outcomes or information on quality of life. This is of particular relevance as this study investigated the change in utilization of mental health care. Consequently we cannot draw clear conclusions on whether changes in utilization resulted in disease-related disadvantages for the patients. Furthermore, the AOK does not have the same market share in all federal states. Hence, the obtained estimates for each district may be more or less representative for the entire population in that district depending on the market share of the AOK. In addition, we chose to aggregate utilization and the strain on intensive care units by month, which might have resulted in imprecise estimates for months in which the incidence of COVID-19 cases changed rapidly. Lastly, we want to note that the stringency index is not available at the state or district level, although the federal states in Germany were largely autonomous in the implemented measures against infections. Consequently, the stringency index was not able to explain any regional variation, even though we assume that diverging containment strategies are responsible for some of the differences between districts.

## Conclusions

This study highlights the reduced utilization of psychiatric and psychosomatic hospital care during the first and second lockdown of the pandemic. On the positive side, we found that psychiatrists in the outpatient sector were mostly able to maintain the level of utilization, although regions with a high burden on intensive care units were more limited than others. The quick adaptation of psychiatrists may have helped to compensate the shortage of inpatient treatment options to some extent.

### Supplementary Information

Below is the link to the electronic supplementary material.

Supplementary file1 (DOCX 690 KB)

## Declarations

### Availability of data and materials

The datasets supporting the conclusions of this article are owned by the German statutory health insurance AOK. Since public deposition of the data would breach ethical and legal compliance, data are only available upon formal request from the research institute of the AOK (WIdO). To request the data, please contact the institutional body of the WIdO (wido@wido.bv.aok.de). To fulfill the legal requirements to obtain that kind of data, researchers must obtain a permission for a specific research question from the German Federal (Social) Insurance Office. Additionally, researchers must conclude a contract with the statutory health insurance regarding data access, which can be requested from the “AOK-Bundesverband GbR” (Federal Association of Local Health Insurance Funds) under http://aok-bv.de/kontakt/. The licensee is permitted to use the data for the purpose of the research proposal within their company, exclusively. Thereby, a company is defined as an economical unit. Licensees are not allowed to pass the data to a third party, or to create software or databases with the exception of scientific publications. Moreover, the study has to be approved by the data protection officer both at the statutory health insurance and the research institute.
