# Supplementary material for: Temporal and spatial changes in the provision of mental health care during the COVID-19 pandemic in Germany: a claims-based cohort study on patients with severe mental disorders
Source: Soc Psychiatry Psychiatr Epidemiol. 2023 Oct 13;59(5):789–97. doi: 10.1007/s00127-023-02571-4 (PMC11087346; doi:10.1007/s00127-023-02571-4)
Supplement: Supplementary file 1 — Supplementary file1 (DOCX 690 KB) [file 127_2023_2571_MOESM1_ESM.docx]

**Supplement:**

| **Table 1: Included and excluded medication-based comorbidity scales** | | |
| --- | --- | --- |
|  | **Chronic condition** | **ATC classification** |
| Excluded | Bone diseases  (osteoporosis) | M05 |
|  | Cancer | L01 |
|  | Dementia | N06D |
|  | Glaucoma | S01E |
|  | Gout, Hyperuricemia | M04 |
|  | HIV | J05AE, J05AG, J05AR |
|  | Intestinal inflammatory diseases | A07EA, A07EC |
|  | Iron deficiency anemia | B03AA, B03AB, B03AC |
|  | Migraines | N02C |
|  | Tuberculosis | J04A |
| Included | Acid related disorders | A02 |
|  | Cardiovascular  diseases (incl.  hypertension) | B01AA, B01AC, C01, C04A, C02, C07, C08, C09 |
|  | Diabetes mellitus | A10A, A10B, A10X |
|  | Epilepsy | N03 |
|  | Hyperlipidemia | C10 |
|  | Pain | N02A, N02B |
|  | Parkinson’s disease | N04, N05B, N05C |
|  | Psycholgical disorders  (sleep disorder,  depression) | N06A |
|  | Psychoses | N05A |
|  | Respiratory illness  (asthma, COPD) | R03 |
|  | Rheumatologic  conditions | M01, M02, L04AA, L04AB |
|  | Thyroid disorders | H03 |
| *Notes:* Medication-based comorbidity scales were excluded if at least one cohort contained less than 30 people in a specific district who were affected by the disease. | | |

**Descriptive Statistics for both cohorts:**

| **Table 2a: Descriptive statistics of the most relevant control variables** | | | |
| --- | --- | --- | --- |
| **Category** | **Outcome** | **Control: N=735,816** | **Pandemic: N=736,972** |
| Socio- demo- graphics | sex (male=0) | 444,360 (60.39%) | 444,394 (60.30%) |
|  | age | 55.44 (17.05) | 55.56 (16.98) |
| Region of residency | Major city | 202,480 (27.52%) | 203,088 (27.56%) |
|  | Smaller city | 270,822 (36.81%) | 270,964 (36.77%) |
|  | Rural area (dense) | 136,980 (18.62%) | 136,776 (18.56%) |
|  | Rural area (sparse) | 125,534 (17.06%) | 126,144 (17.12%) |
| Diagnosis at baseline | Bipolar disorder | 34,053 (4.63%) | 34,345 (4.66%) |
|  | Multiple diagnoses | 75,214 (10.22%) | 75,093 (10.19%) |
|  | Severe depression | 339,414 (46.13%) | 343,161 (46.56%) |
|  | Personality disorder | 136,012 (18.48%) | 134,838 (18.30%) |
|  | Schizophrenia | 125,709 (17.08%) | 124,200 (16.85%) |
|  | Schizoaffective disorder | 25,414 (3.45%) | 25,335 (3.44%) |
| Source of diagnosis | Outpatient clinic | 100,310 (13.63%) | 102,214 (13.87%) |
|  | Psychiatric clinic | 35,916 (4.88%) | 35,702 (4.84%) |
|  | Outpatient provider | 287,116 (39.02%) | 287,356 (38.99%) |
|  | Mental health specialist | 312,474 (42.47%) | 311,700 (42.29%) |
| Utilization during the preperiod | Antidepressants (ddd) | 148.83 (228.43) | 151.09 (230.69) |
|  | Antipsychotics (ddd) | 90.41 (228.31) | 89.26 (224.25) |
|  | Psychiatrist visits | 2.58 (6.70) | 2.53 (6.46) |
|  | Hospital days | 3.99 (17.13) | 4.15 (17.82) |
| All numbers were rounded to two decimal places. We report the number of cases with a particular feature for categorical variables (percentage in parentheses) and the mean for all interval-scaled variables (standard deviation in parentheses) | | | |

| **Table 2b: Descriptive statistics of the region of residency** | | | |
| --- | --- | --- | --- |
| **Category** | **Outcome** | **Control: N=735,816** | **Pandemic: N=736,972** |
| Socio- demo- graphics | Baden-Württemberg | 114,954 (15.62%) | 114,412 (15.52%) |
|  | Bavaria | 128,061 (17.40%) | 129,498 (17.57%) |
|  | Berlin | 25,800 (3.51%) | 24,742 (3.36%) |
|  | Brandenburg | 18,159 (2.47%) | 17,780 (2.41%) |
|  | Bremen | 7,201 (0.98%) | 7,401 (1.00%) |
|  | Hamburg | 12,699 (1.73%) | 12,654 (1.72%) |
|  | Hesse | 42,876 (5.83%) | 43,194 (5.86%) |
|  | Mecklenburg Western Pomerania | 13,573 (1.84%) | 13,266 (1.80%) |
|  | Lower Saxony | 71,712 (9.75%) | 72,387 (9.82%) |
|  | Northrhine-Westphalia | 149,702 (20.35%) | 151,373 (20.54%) |
|  | Rhineland Palatinate | 32,730 (4.45%) | 32,003 (4.34%) |
|  | Saarland | 6,832 (0.93%) | 6,788 (0.92%) |
|  | Saxony | 44,758 (6.08%) | 44,539 (6.04%) |
|  | Saxony-Anhalt | 19,372 (2.63%) | 19,371 (2.63%) |
|  | Schleswig Holstein | 20,725 (2.82%) | 20,988 (2.85%) |
|  | Thuringia | 26,662 (3.62%) | 26,576 (3.61%) |
| All numbers were rounded to two decimal places. We report the number of cases with a particular feature for categorical variables (percentage in parentheses) and the mean for all interval-scaled variables (standard deviation in parentheses) | | | |

| **Table 2c: Descriptive statistics of the medication-based comorbidity scale** | | | |
| --- | --- | --- | --- |
| **Category** | **Outcome** | **Control: N=735,816** | **Pandemic: N=736,972** |
| Medication-based comorbidity scale | Acid related disorders | 349,694 (47.52%) | 342,444 (46.47%) |
|  | Bone diseases  (osteoporosis) | 13,147 (1.79%) | 13,189 (1.79%) |
|  | Cancer | 13,968 (1.90%) | 13,661 (1.85%) |
|  | Cardiovascular  diseases (incl.  hypertension) | 464,413 (63.12%) | 464,794 (63.07%) |
|  | Dementia | 11,198 (1.52%) | 10,681 (1.45%) |
|  | Diabetes mellitus | 104,140 (14.15%) | 106,132 (14.40%) |
|  | Epilepsy | 106,864 (14.52%) | 107,978 (14.65%) |
|  | Glaucoma | 23,578 (3.20%) | 23,590 (3.20%) |
|  | Gout, Hyperuricemia | 34,153 (4.64%) | 33,509 (4.55%) |
|  | HIV | 1,618 (0.22%) | 1,732 (0.24%) |
|  | Hyperlipidemia | 130,056 (17.68%) | 135,151 (18.34%) |
|  | Intestinal inflammatory diseases | 5,632 (0.77%) | 5,693 (0.77%) |
|  | Iron deficiency anemia | 19,792 (2.69%) | 20,134 (2.73%) |
|  | Migraines | 10,902 (1.48%) | 11,713 (1.59%) |
|  | Pain | 200,600 (27.26%) | 204,592 (27.76%) |
|  | Parkinson’s disease | 166,584 (22.64%) | 161,290 (21.89%) |
|  | Psycholgical disorders  (sleep disorder,  depression) | 362,338 (49.24%) | 365,760 (49.63%) |
|  | Psychoses | 293,944 (39.95%) | 295,289 (40.07%) |
|  | Respiratory illness  (asthma, COPD) | 100,799 (13.70%) | 102,328 (13.88%) |
|  | Rheumatologic  conditions | 221,683 (30.13%) | 221,014 (29.99%) |
|  | Thyroid disorders | 194,803 (26.47%) | 197,251 (26.77%) |
|  | Tuberculosis | 395 (0.05%) | 451 (0.06%) |
| All numbers were rounded to two decimal places. We report the number of cases with a particular feature for categorical variables (percentage in parentheses) and the mean for all interval-scaled variables (standard deviation in parentheses) | | | |

**Figure 1: Data flowchart**


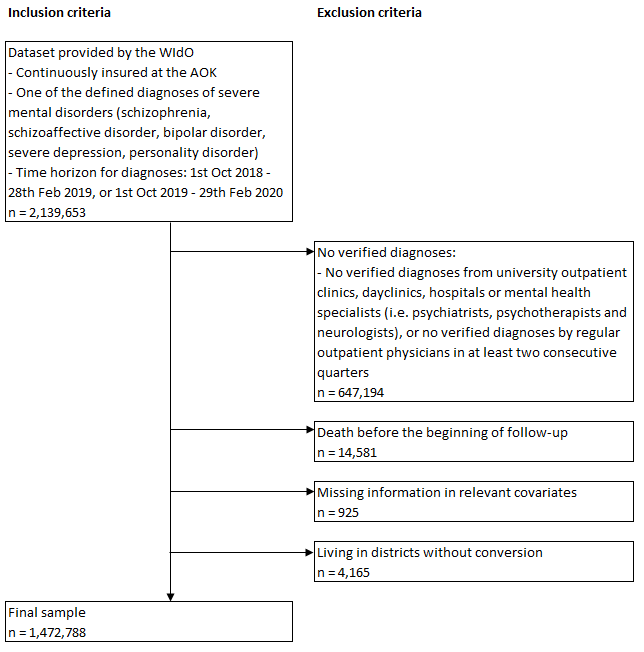


*Notes:* Number of patients included or excluded and corresponding criteria.

**Modelling results:**

We assumed the absence of spatial autocorrelation in all meta-analytic models. Figure 1 shows the results of the formal test for autocorrelation using Moran’s I.

**Figure 2: Moran’s I for each calendar month**

**
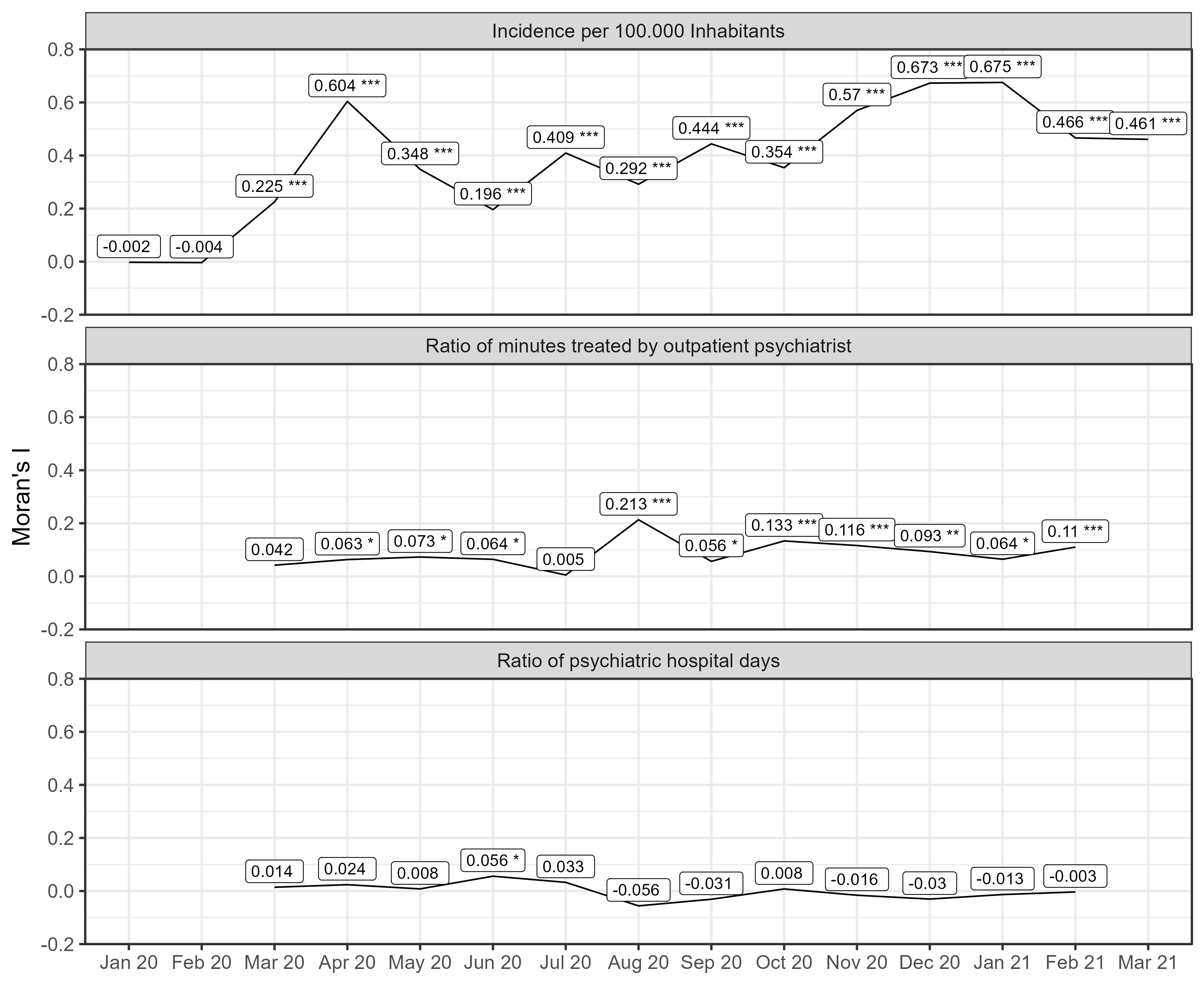
**

*Notes:* The upper panel shows Moran’s I for the average 7 day incidence rate of COVID-19 cases. It was included as a reference outcome to demonstrate the amount of autocorrelation that would be expected for spatially dependent outcomes. The lower two panels depict Moran’s I by calender month for utilization changes in the inpatient sector (i.e. psychiatric hospital days) and the outpatient sector (i.e. minutes treated by psychiatrists)

| **Table 3a:** **Results of the metaanalytic model to explain changes in psychiatric hospital days** | | | |
| --- | --- | --- | --- |
| **Predictor** | **β** |  | **Standard error** |
| Intercept | 0.028 |  | (0.315) |
| Lockdown 1 (L1) | -0.506 |  | (0.358) |
| Lockdown 2 (L2) | 1.128 | ** | (0.379) |
| Stringency index | -0.008 | *** | (0.001) |
| Intensive care unit cases per 100.000 people | 0.000 |  | (0.004) |
| German index of social deprivation | 0.016 |  | (0.015) |
| Hospitals per 100.000 people | 0.015 |  | (0.022) |
| Psychiatrists per 100.000 people | 0.001 |  | (0.003) |
| General practitioners per 100.000 people | 0.001 |  | (0.002) |
| Average age | 0.004 |  | (0.007) |
| Population density | 0.000 |  | (0.000) |
| Intensive care unit cases per 100.000 people in L1 | -0.015 |  | (0.011) |
| Intensive care unit cases per 100.000 people in L2 | -0.008 |  | (0.006) |
| German index of social deprivation in L1 | -0.002 |  | (0.017) |
| German index of social deprivation in L2 | 0.017 |  | (0.018) |
| Hospitals per 100.000 people in L1 | -0.010 |  | (0.025) |
| Hospitals per 100.000 people in L2 | 0.008 |  | (0.026) |
| Psychiatrists per 100.000 people in L1 | 0.007 |  | (0.004) |
| Psychiatrists per 100.000 people in L2 | -0.008 |  | (0.004) |
| General practitioners per 100.000 people in L1 | 0.002 |  | (0.002) |
| General practitioners per 100.000 people in L2 | 0.004 |  | (0.002) |
| Average age in L1 | 0.006 |  | (0.008) |
| Average age in L2 | -0.026 | ** | (0.008) |
| Population density in L1 | 0.000 |  | (0.000) |
| Population density in L2 | 0.000 |  | (0.000) |
| N=1,472,788, *p<.05, **p<.01, ***p<.001, L1 refers to the first lockdown period from March 2020 until May 2020, L2 refers to the second lockdown period from December 2020 until February 2021, we included interaction effects for each control variable and the main predictors to allow for deviating effects in the two lockdown periods. | | | |

| **Table 3b:** **Results of the metaanalytic model to explain changes in minutes treated by psychiatrists** | | | |
| --- | --- | --- | --- |
| **Predictor** | **β** |  | **Standard error** |
| Intercept | 0.591 | *** | (0.141) |
| Lockdown 1 (L1) | -0.216 |  | (0.214) |
| Lockdown 2 (L2) | -0.008 |  | (0.220) |
| Stringency index | -0.005 | *** | (0.000) |
| Intensive care unit cases per 100.000 people | 0.002 |  | (0.002) |
| German index of social deprivation | -0.009 |  | (0.007) |
| Hospitals per 100.000 people | -0.011 |  | (0.010) |
| Psychiatrists per 100.000 people | -0.001 |  | (0.002) |
| General practitioners per 100.000 people | -0.002 | ** | (0.001) |
| Average age | -0.003 |  | (0.003) |
| Population density | 0.000 |  | (0.000) |
| Intensive care unit cases per 100.000 people in L1 | -0.015 | * | (0.006) |
| Intensive care unit cases per 100.000 people in L2 | -0.018 | *** | (0.003) |
| German index of social deprivation in L1 | -0.009 |  | (0.010) |
| German index of social deprivation in L2 | -0.010 |  | (0.011) |
| Hospitals per 100.000 people in L1 | 0.007 |  | (0.016) |
| Hospitals per 100.000 people in L2 | -0.030 |  | (0.016) |
| Psychiatrists per 100.000 people in L1 | 0.002 |  | (0.002) |
| Psychiatrists per 100.000 people in L2 | 0.001 |  | (0.002) |
| General practitioners per 100.000 people in L1 | -0.001 |  | (0.001) |
| General practitioners per 100.000 people in L2 | 0.001 |  | (0.001) |
| Average age in L1 | 0.002 |  | (0.005) |
| Average age in L2 | 0.003 |  | (0.005) |
| Population density in L1 | 0.000 |  | (0.000) |
| Population density in L2 | 0.000 |  | (0.000) |
| N=1,472,788, *p<.05, **p<.01, ***p<.001, L1 refers to the first lockdown period from March 2020 until May 2020, L2 refers to the second lockdown period from December 2020 until February 2021, we included interaction effects for each control variable and the main predictors to allow for deviating effects in the two lockdown periods. | | | |

**Modelling predictions:**

To illustrate the size of the effects, we used the models described in table 2a and 2b to predict utilization changes as a function of the most relevant predictors (i.e. main and interaction effects of intensive care unit cases per 100,000 people, the stringency index and the first and second lockdown). To obtain a realistic range of intensive care unit cases per month, we calculated the first, second and third quartile by month. However, the stringency index was set to a fixed value – based on the actual value assigned to Germany as a whole during that month. Hence, we would expect three different predictions based on the respective quartile in intensive care unit cases. If fewer predictions were obtained, it is because two or more quartiles were identical during that month.

**Figure 3: Predicted ratio of psychiatric hospital days as a function of the inputs**

**
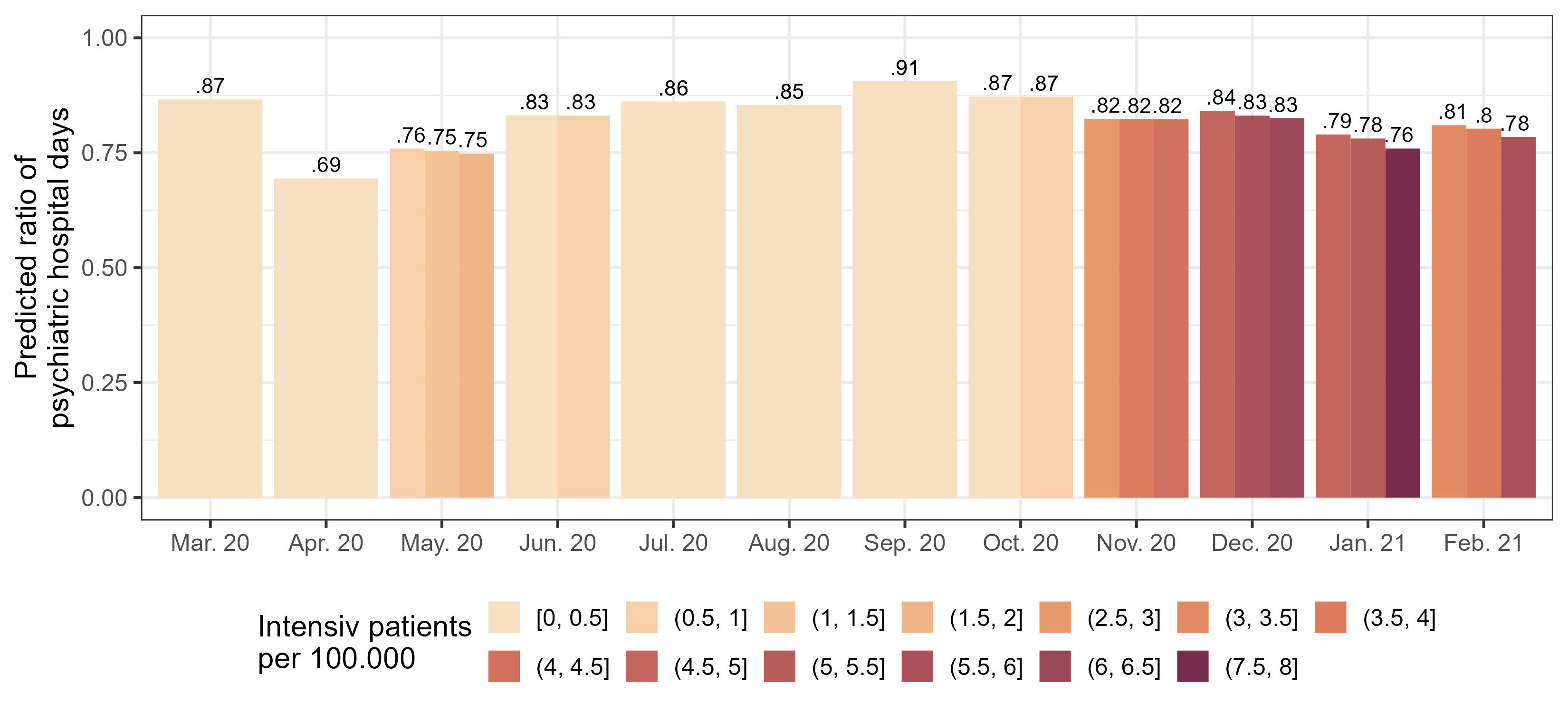
**

*Notes:* We used the model described in table 2a to estimate the ratios. All control variables were set to their mean. To allow easier assignments of colours, we categorized the various quartiles in intensive care by splitting the continuous range of values into intervals with a length of 0.5

**Figure 4: Predicted ratio of minutes treated by psychiatrists as a function of the inputs**

**
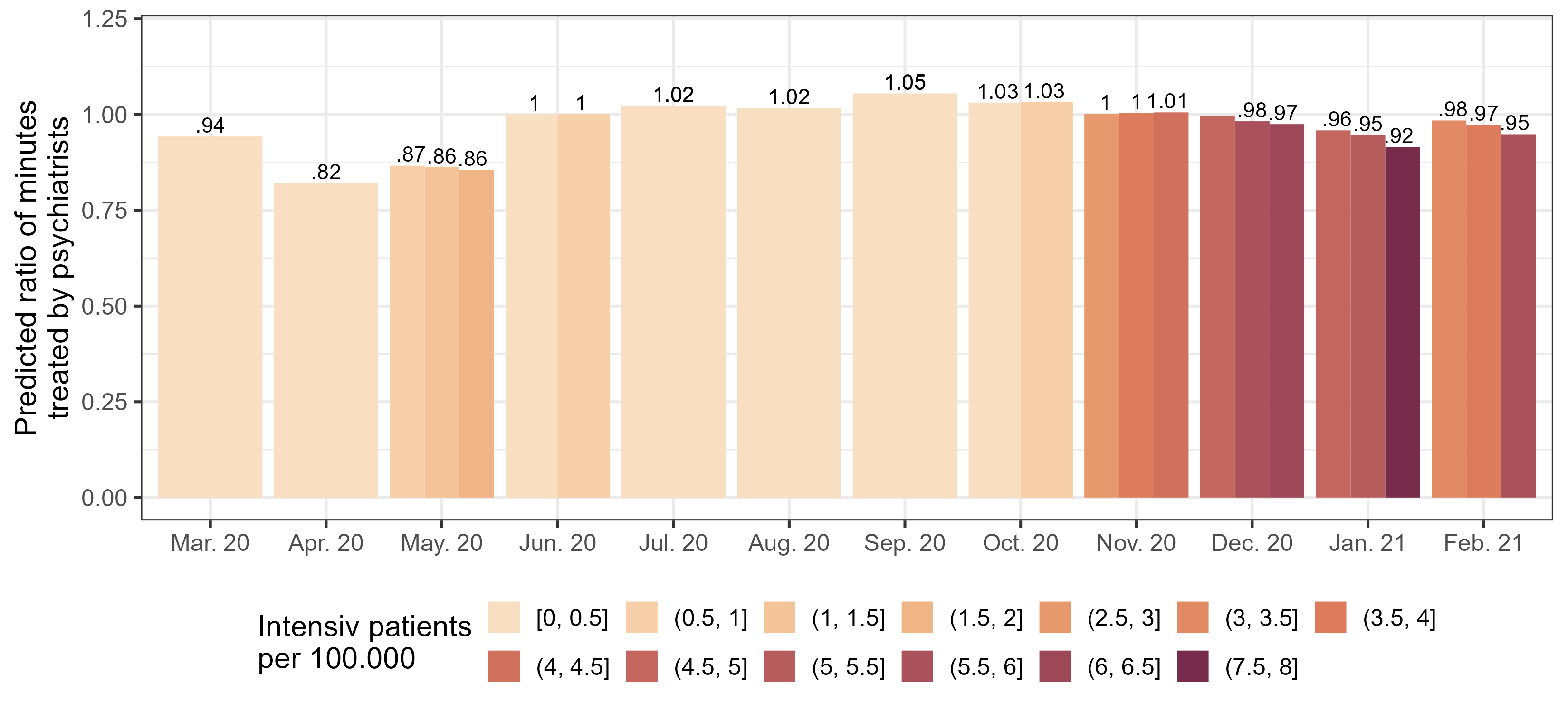
**

*Notes:* We used the model described in table 2b to estimate the ratios. All control variables were set to their mean. To allow easier assignments of colours, we categorized the various quartiles in intensive care by splitting the continuous range of values into intervals with a length of 0.5
